# Supplementary material for: A systematic review of the direct and indirect effects of herbivory on plant reproduction mediated by pollination
Source: PeerJ. 2020 Jun 8;8:e9049. doi: 10.7717/peerj.9049 (PMC7289145; doi:10.7717/peerj.9049)
Supplement: Supplemental Information 1 — For specific association with a given study, see Table A2. [file peerj-08-9049-s001.docx]

**Supplemental Table S1**

List of all pollinator, herbivore, and plant species included in the review. For specific association with a given study, see table A2.

| **Type** | **Class** | **Order** | **Family** | **Genus** | **Species** |
| --- | --- | --- | --- | --- | --- |
| plant | Dicotyledons | Apiales | Apiaceae | *Eryngium* | *yuccifolium* |
| plant | Dicotyledons | Apiales | Apiaceae | *Pastinaca* | *sativa* |
| plant | Dicotyledons | Asterales | Asteraceae | *Cardus* | *thoermeri* |
| plant | Dicotyledons | Asterales | Asteraceae | *Centaurea* | *solstitialis* |
| plant | Dicotyledons | Asterales | Asteraceae | *Rudbeckia* | *hirta* |
| plant | Dicotyledons | Brassicales | Cleomaceae | *Isomeris* | *arborea* |
| plant | Dicotyledons | Capparales | Brassicaceae | *Brassica* | *napus* |
| plant | Dicotyledons | Capparales | Brassicaceae | *Brassica* | *nigra* |
| plant | Dicotyledons | Capparales | Brassicaceae | *Brassica* | *rapa* |
| plant | Dicotyledons | Capparales | Brassicaceae | *Lepidium* | *papilliferum* |
| plant | Dicotyledons | Capparales | Brassicaceae | *Raphanus* | *raphanistrum* |
| plant | Dicotyledons | Cornales | Loasaceae | *Loasa* | *tricolor* |
| plant | Dicotyledons | Cucurbitales | Cucurbitaceae | *Cucumis* | *sativus* |
| plant | Dicotyledons | Euphorbiales | Euphorbiaceae | *Cnidoscolus* | *acontifolius* |
| plant | Dicotyledons | Fabales | Fabaceae | *Centrosema* | *virginianum* |
| plant | Dicotyledons | Fabales | Fabaceae | *Prosopis* | *flexuosa* |
|  |  |  |  |  |  |
| plant | Dicotyledons | Gentianales | Asclepiadaceae | *Cynanchum* | *diemii* |
| plant | Dicotyledons | Geraniales | Balsaminaceae | *Impatiens* | *capensis* |
| plant | Dicotyledons | Malvales | Elaeocarpaceae | *Aristotelia* | *chilensis* |
| plant | Dicotyledons | Myrtales | Onagraceae | *Oenothera* | *macrocarpa* |
| plant | Dicotyledons | Myrtales | Onagraceae | *Clarkia* | *xantiana ssp. xantiana* |
| plant | Dicotyledons | Ranunculales | Berberidaceae | *Berberis* | *darwinii* |
| plant | Dicotyledons | Rosales | Grossulariaceae | *Ribes* | *magellanicum* |
| plant | Dicotyledons | Rubiales | Rubiaceae | *Bouvardia* | *ternifolia* |
| plant | Dicotyledons | Santalales | Loranthaceae | *Tristerix* | *aphyllus* |
| plant | Dicotyledons | Santales | Loranthaceae | *Peraxilla* | colensoi |
| plant | Dicotyledons | Santales | Loranthaceae | *Peraxilla* | *Tetrapetala* |
| plant | Dicotyledons | Scrophulariaceae | Scrophulariaceae | *Castilleja* | *indivisa* |
| plant | Dicotyledons | Scrophulariales | Acanthaceae | *Ruellia* | *nudiflora* |
| plant | Dicotyledons | Scrophulariales | Scrophulariaceae | *Linaria* | *dalmatica* |
| plant | Dicotyledons | Scrophulariales | Scrophulariaceae | *Mimulus* | *aurantiacus* |
| plant | Dicotyledons | Scrophulariales | Scrophulariaceae | *Pedicularis* | *gruina* |
| plant | Dicotyledons | Scrophulariales | Scrophularicaeae | *Verbascum* | *nigrum* |
| plant | Dicotyledons | Solanales | Hydrophyllaceae | *Nemophila* | *menziesii* |
| plant | Dicotyledons | Solanales | Polemoniaceae | *Ipomopsis* | *aggregata* |
| plant | Dicotyledons | Solanales | Polemoniaceae | *Ipomopsis* | *aggregata* subssp*. Candida* |
| plant | Dicotyledons | Violales | Cistaceae | *Halimium* | *halimifolium* |
| plant | Dicotyledons | Violales | Cucurbitaceae | *Cucumis* | *melo* |
| plant | Dicotyledons | Violales | Cucurbitaceae | *Cucurbita* | *moschata* |
| plant | Monocotyledons | Asparagales | Orchidaceae | *Myrmecophila* | *tibicinis* |
| plant | Monocotyledons | Bromeliales | Bromeliaceae | *Aechmea* | *pectinata* |
| plant | Monocotyledons | Geonomeae | Arecaceae | *Calyptrogyne* | *ghiesbreghtiana* |
| plant | Monocotyledons | Liliales | Liliaceae | *Alstroemeria* | *aurea* |
| plant | Monocotyledons | Liliales | Liliaceae | *Alstroemeria* | *ligtu va. Simsii* |
| plant | Monocotyledons | Liliales | Liliaceae | *Alstroemeria* | *umbellata* |
| plant | Monocotyledons | Liliales | Liliaceae | *Trillium* | *grandiflorum* |
| plant | Monocotyledons | Liliales | Pontederiaceae | *Eichhornia* | *crassipes* |
| plant | Monocotyledons | Orchidales | Orchidaceae | *Platanthera* | *bifolia* |
| plant | Monocotyledons | Zingiberales | Heliconiaceae | *Heliconia* | *spathocircinata* |
| herbivore | Insecta | Coleoptera | Chrysomelidae | *Acalymma* | *vitattum* |
| herbivore | Insecta | Coleoptera | Chrysomelidae | *Phyllotreta* | sp. |
| herbivore | Insecta | Coleoptera | Curculionidae | *Cionus* | *nigritarsis* |
| herbivore | Insecta | Coleoptera | Curculionidae | *Mecinus* | *janthiniformis* |
| herbivore | Insecta | Coleoptera | Meligethes | *Meligethes* | *aeneus* |
| herbivore | Insecta | Coleoptera | Nitidulidae | *Meligethes* | *rufimanus* |
| herbivore | Insecta | Diptera | Anthomyiidae | *Delia* | *radicum* |
| herbivore | Insecta | Hemiptera | Aphididae | *Aphis* | *alstroemeriae* |
| herbivore | Insecta | Hemiptera | Aphididae | *Brachycaudus* | *cardui* |
| herbivore | Insecta | Hemiptera | Aphididae | *Brevicoryne* | *brassicae* |
| herbivore | Insecta | Hemiptera | Aphididae | *Lipaphis* | *erysimi* |
| herbivore | Insecta | Homoptera | Aphrophoridae | *Philaenus* | *spumarius* |
| herbivore | Insecta | Hymenoptera | Tenthredinidae | *Athalia* | *rosae* |
| herbivore | Insecta | Lepidoptera | Gelechiidae | *Coleotechnites* | *eryngiella* |
| herbivore | Insecta | Lepidoptera | Noctuidae | *Helicoverpa* | *armigera* |
| herbivore | Insecta | Lepidoptera | Noctuidae | *Mamestra* | *brassicae* |
| herbivore | Insecta | Lepidoptera | Noctuidae | *Spodoptera* | *littoralis* |
| herbivore | Insecta | Lepidoptera | Oecophoridae | *Depressaria* | *pastinacella* |
| herbivore | Insecta | Lepidoptera | Pieridae | *Pieris* | *brassicae* |
| herbivore | Insecta | Lepidoptera | Pieridae | *Pieris* | *rapae* |
| herbivore | Insecta | Lepidoptera | Plutellidae | *Plutella* | *xylostella* |
| herbivore | Insecta | Orthoptera | Tettgoniidae | *Tettgoniidae* | spp. |
| herbivore | Malacostraca | Decapoda | Grapsidae | *Armases* | *angustipes* |
| herbivore | Mammalia | Artiodactyla | Bovidae | *Bos* | *taurus* |
| herbivore | Mammalia | Artiodactyla | Bovidae | *Ovis* | *aries* |
| herbivore | Mammalia | Artiodactyla | Cervidae | *Odocoileus* | *virginianus* |
| herbivore | Mammalia | Perissodactyla | Equidae | *Equus* | *ferus* |
| pollinator | Aves | Apodiformes | Trochilidae | *Phaethornis* | *ruber* |
| pollinator | Aves | Apodiformes | Trochilidae | *Ramphodon* | *naevius* |
| pollinator | Aves | Apodiformes | Trochilidae | *Thalurania* | *glaucopis* |
| pollinator | Insecta | Coleoptera |  |  | spp. |
| pollinator | Insecta | Diptera | Syrphidae | *Episyrphus* | *balteatus* |
| pollinator | Insecta | Diptera | Syrphidae |  | spp. |
| pollinator | Insecta | Hymenoptera |  |  | spp. |
| pollinator | Insecta | Hymenoptera | Apidae | *Apis* | *mellifera* |
| pollinator | Insecta | Hymenoptera | Apidae | *Bombus* | *pensylvanicus* |
| pollinator | Insecta | Hymenoptera | Apidae | *Bombus* | spp. |
| pollinator | Insecta | Hymenoptera | Apidae | *Bombus* | *terrestris* |
| pollinator | Insecta | Hymenoptera | Apidae | *Melissodes* | spp. |
| pollinator | Insecta | Hymenoptera | Apidae | *Xylocopa* | *micans* |
| pollinator | Insecta | Hymenoptera | Megachilidae | *Megachile* | spp. |
| pollinator | Insecta | Lepidoptera |  |  | spp. |
| pollinator | Insecta | Lepidoptera | Nymphalidae | *Speyeria* | *idalia* |
| pollinator | Insecta | Lepidoptera | Pieridae | *Pieris* | *brassicae* |
| pollinator | Mammalia | Chiroptera | Glossophaginae | *Glossophaga* | spp. |
