# Supplementary material for: A systematic review of the direct and indirect effects of herbivory on plant reproduction mediated by pollination
Source: PeerJ. 2020 Jun 8;8:e9049. doi: 10.7717/peerj.9049 (PMC7289145; doi:10.7717/peerj.9049)
Supplement: Supplemental Information 4 [file peerj-08-9049-s004.docx]

Supplemental File: PRISMA Documentation

This systematic review was conducted to not only synthesize the contemporary literature on the effects of herbivores on plant reproduction as mediated by pollinators, but also to explore how different forms of herbivory can effect this change. While the idea that herbivores and pollinators interact to impact plant reproduction is not new, reviews of the relative importance of direct and indirect damage to plants as it relates to pollination are lacking. In this review, we provide an updated synthesis on herbivore-pollinator interactions, as well as a synthesis of the frequency and conclusions that can be made on the relative importance of direct and indirect damage to floral tissue on pollination and plant reproduction.
