# Supplementary material for: A systematic review of the direct and indirect effects of herbivory on plant reproduction mediated by pollination
Source: PeerJ. 2020 Jun 8;8:e9049. doi: 10.7717/peerj.9049 (PMC7289145; doi:10.7717/peerj.9049)
Supplement: Supplemental Information 5 [file peerj-08-9049-s005.docx]

| **Supplemental Data File 1** | outlining of each treatment for each study |
| --- | --- |
| article id | unique id corresponding to each article. Corresponds to articles in Supplemental Data File 2 |
| study year | Year for that analysis, in the case of an analysis over multiple years, the final year is recorded |
| Plant Genus | Plant genus used in the analysis |
| Plant Species | Plant species used in the analysis |
| Herbivore Species | Herbivore species used in the analysis |
| Pollinator Species | Pollinator species used in the analysis |
| Low pollination method | The way that no, or little pollination was achieved compared to the high pollination method. Values include: no pollination, natural, exclosure (pollinators excluded manually). |
| High pollination method | The way that pollination was increased or supplemental compared to the low pollination method. Values include artificial supplemental (hand pollination), pollinator addition, natural. |
| Low herbivory method | The way that no, or little herbivory was achieved compared to the high herbivory method. Values include pesticide, natural, no herbivory, exclosure. In the case where two herbivores or two types of herbivory are compared, the herbivore species or type that was found to have a less detrimental effect is listed instead. |
| High herbivory method | The way that herbivory was increased compared to the low herbivory method. Values include natural, defense removal, herbivore addition, artificial herbivory. In the case where two herbivores or two types of herbivory are compared, the herbivore species or type that was found to have a more detrimental effect is listed instead. |
| Herbivory Type | Type of herbivory, e.g. folivory, florivory… |
| Indirect Response Variable | In the case of when herbivory or pollination has an indirect effect on a response variable, the variable that mediates the response is indicated (e.g. if herbivory decreases flower number, which in turn decreases pollinator visitation, flower number would be the variable indicated). If no variable is indicated, the effect is direct. Generally used for interpreting path analyses. |
| Response | Response variable for that analysis |
| Response Category | Response variable category - either floral expression, pollination, or seed production. Floral expression represents response variables that are expressed in the quality of floral display (generally quantity or quality of flowers), pollination represents response variables that expressly involve pollination (often by a pollinator), while seed production represents response variables that are specifically measured post-fertilization (I.e. seed set and fruit set). |
| Effect | Direction of effect of increasing the treatment (e.g. negative for a herbivory treatment implies increased herbivory had a negative effect or decreased herbivory had a positive effect on the response variable). For interaction effects "positive" is recorded to indicate that there is an interaction but not direction. While in many studies multiple measures of a single category may have been presented (e.g. seed number and seed weight both represent seedset), if any of these measures were statistically significant, a negative or positive response is reported here. |
|  |  |
| **Supplemental Data File 2** | Information about each publication used - including identifiers of the article and study info |
| Article id | Unique article id |
| author | list of authors |
| title | article title |
| journal | journal published in |
| publication year | year article was published |
| volume | journal volume |
| issue | volume issue |
| page | starting page number |
| country | country of study |
| state/province | the state or province the study happened in |
| location | more specific location than state or province (e.g. city, county, park) |
| lat | latitude provided of study location (or average latitude of multiple sites) |
| long | longitude provided of study location (or average longitude of multiple sites) |
| greenhouse experiment | was all or part of the experiment done in a greenhouse? |
| number of sites | number of sites that were used for the study |
| start year | starting year of the study |
| end year | final year of the study |
| number of years | number of years in which data was collected or study actively carried out |
| habitat1 | habitat description |
| habitat2 | habitat description |
| habitat3 | habitat description |
| Number of Plant taxa | number of plant taxa included in the study (generally number of species). “Community” refers to the entire community and not a specific number of taxa. |
| Number of herbivore taxa | number of herbivore taxa included in the study (generally number of species, but sometimes higher levels such as genus or family). “Community” refers to the entire community and not a specific number of taxa. |
| Number of pollinator taxa | number of pollinator taxa included in the study (generally number of species, but sometimes higher levels such as genus or family). 100 indicates the entire community |
|  |  |
|  |  |
|  |  |
|  |  |
|  |  |
|  |  |
|  |  |
|  |  |
|  |  |
|  |  |
