# Supplementary material for: A systematic review of the direct and indirect effects of herbivory on plant reproduction mediated by pollination
Source: PeerJ. 2020 Jun 8;8:e9049. doi: 10.7717/peerj.9049 (PMC7289145; doi:10.7717/peerj.9049)
Supplement: Supplemental Information 6 [file peerj-08-9049-s006.doc]

**Supplemental Table S2**

List of authors with their identification as well as a list of all plant, herbivore, and pollinator species examined. The type(s) of herbivory utilized is also provided.

| **Author** | **Plant Species** | **Herbivore Species** | **Pollinator Species** | **Herbivory Types** |
| --- | --- | --- | --- | --- |
| Adler, 2000 | Scrophulariaceae *Castilleja indivisa* |  |  | florivory |
| Adler, Karban & Strauss, 2001 | Scrophulariaceae *Castilleja indivisa* |  |  | florivory |
| Agrawal et al., 2000 | Asteraceae *Centaurea solstitialis* |  |  | open |
| Åhman, Lehrman & Ekbom, 2009 | Brassicaceae *Brassica napus* | *Meligethes aeneus* | *Bombus terrestris* | florivory |
| Arceo-Gómez, Parra-Tabla & Navarro, 2009 | Euphorbiaceae *Cnidoscolus acontifolius* |  |  | folivory |
| Aschero & Vázquez, 2009 | Fabaceae *Prosopis flexuosa* | *Bos taurus* |  | grazing |
| Barber et al., 2015 | Cucurbitaceae *Cucumis sativus* | *Acalymma vitattum* |  | root herbivory |
| Benning & Moeller, 2019 | Onagraceae *Clarkia xantiana* ssp. *xantiana* |  |  | open |
| Botto-Mahan et al., 2011 | Liliaceae *Alstroemeria ligtu va. Simsii* |  |  | florivory |
| Brody & Irwin, 2012 | Polemoniaceae *Ipomopsis aggregata* |  |  | grazing |
|  |  |  |  |  |
| Buchanan, 2014 | Pontederiaceae *Eichhornia crassipes* |  |  | folivory, stem damage |
| Caballero et al., 2013 | Loranthaceae *Tristerix aphyllus* | Formicidae spp. | *Sephanoides sephanoides* | florivory |
| Canela & Sazima, 2003 | Bromeliaceae *Aechmea pectinata* | *Armases angustipes* | Trochilidae spp. | florivory |
| Cardel & Koptur, 2010 | Fabaceae *Centrosema virginianum* |  | *Melissodes spp, Megachile spp, Bombus pensylvanicus, Xylocopa micans* | florivory |
| Cares-Suárez et al., 2011 | Loasaceae *Loasa tricolor* |  |  | florivory |
| Cariveau & Norton, 2014 | Scrophulariaceae *Linaria dalmatica* | *Mecinus janthiniformis* |  | stem damage |
| Chalcoff, Lescano & Devegili, 2019 | Asteraceae *Cardus thoermeri* | *Brachycaudus cardui* |  | stem damage |
| Cunningham, 1995 | Arecaceae *Calyptrogyne ghiesbreghtiana* |  | *Glossophaga spp* | florivory |
| Danderson & Molano-Flores, 2010 | Apiaceae *Eryngium yuccifolium* | *Coleotechnites eryngiella* |  | florivory |
| Enri et al., 2017 |  | *Bos taurus, Ovis aries* |  | grazing |
| Garrido et al., 2019 |  | *Equus ferus* | Lepidoptera spp., *Bombus* spp. | grazing |
| Soper Gorden & Adler, 2016 | Balsaminaceae *Impatiens capensis* |  |  | florivory |
| Hambäck, 2001 | Asteraceae *Rudbeckia hirta* | *Philaenus spumarius* |  | stem damage |
| Hladun & Adler, 2009 | Cucurbitaceae *Cucurbita moschata* | *Acalymma vittatum* |  | root herbivory, folivory |
|  |  |  |  |  |
| Juenger & Bergelson, 1997 | Polemoniaceae *Ipomopsis aggregata subssp. Candida* |  |  | grazing |
| Kelly et al., 2008 | Loranthaceae *Peraxilla colensoi,* Loranthaceae *Peraxilla tetrapetala* | *Zelleria maculata* |  | florivory |
| Knight, 2004 | Liliaceae *Trillium grandiflorum* | *Odocoileus virginianus* |  | grazing |
| Krupnick & Weis, 1999 | Cleomaceae *Isomeris arborea* | *Meligethes rufimanus* |  | florivory |
| Krupnick, Weis & Campbell, 1999 | Cleomaceae *Isomeris arborea* | *Meligethes rufimanus* |  | florivory |
| Leavitt & Robertson, 2006 | Brassicaceae *Lepidium papilliferum* | *Phyllotreta sp.* |  | florivory |
| Liao et al., 2013 | Scrophulariaceae *Pedicularis gruina* | *Helicoverpa armigera* | *Bombus* spp. | florivory |
| Lohman, Zangerl & Berenbaum, 1996 | Apiaceae *Pastinaca sativa* | *Depressaria pastinacella* |  | florivory |
| Louthan et al., 2019 |  | Mammalia spp. |  | grazing |
| Lucas-Barbosa et al., 2016 | Brassicaceae *Brassica nigra* | *Pieris brassicae* | *Episyrphus balteatus, Pieris brassicae* | florivory, folivory |
| Lucas-Barbosa et al., 2013 | Brassicaceae *Brassica nigra* | *Pieris brassicae* | *Bombus spp, Apis mellifera* | florivory, folivory |
| McCall, 2010 | Hydrophyllaceae *Nemophila menziesii* |  |  | florivory |
| Missagia & Alves, 2017 | Heliconiaceae *Heliconia spathocircinata* | Diptera spp. | *Phaethornis ruber, Ramphodon naevius, Thalurania glaucopis* | florivory |
| Moranz, Fuhlendorf & Engle, 2014 |  | *Bos taurus* | *Speyeria idalia* | grazing |
| Mothershead & Marquis, 2000 | Onagraceae *Oenothera macrocarpa* |  |  | florivory, folivory |
| Motzke et al., 2015 | Cucurbitaceae *Cucumis sativus* |  |  | folivory |
| Munguía-Rosas et al., 2015 | Acanthaceae *Ruellia nudiflora* |  |  | folivory |
| Sasal, Farji-Brener & Raffaele, 2017 | Berberidaceae *Berberis darwinii* | *Bos taurus* |  | grazing, folivory |
| Rusman et al., 2019 | Brassicaceae *Brassica nigra* | *Athalia rosae, Plutella xylostella, Brevicoryne brassicae, Lipaphis erysimi, Delia radicum* | *Episyrphus balteatus, Pieris brassicae* | stem damage, root herbivory, folivory |
| Scopece, Frachon & Cozzolino, 2019 | Asteraceae *Cardus thoermeri* | *Pieris rapae, Mamestra brassicae, Spodoptera littoralis* | *Apis mellifera* | folivory |
| Sõber, Moora & Teder, 2010 | Scrophulariaceae *Verbascum nigrum* | *Cionus nigritarsis* |  | florivory |
| Sõber, Teder & Moora, 2009 | Scrophulariaceae *Verbascum nigrum* | *Cionus nigritarsis* |  | florivory |
| Strauss & Murch, 2004 | Cucurbitaceae *Cucumis melo* |  |  | folivory |
| Suárez, Gonzáles & Gianoli, 2009 | Liliaceae *Alstroemeria ligtu va. Simsii* |  |  | folivory |
| Suárez-Esteban, Delibes & Fedriani, 2014 | Cistaceae *Halimium halimifolium* |  |  | grazing |
| Sutter & Albrecht, 2016 | *Brassicaceae Brassica napus* | *Meligethes aeneus* |  | florivory |
| Torres et al., 2008 | Rubiaceae *Bouvardia ternifolia* |  |  | folivory |
| Tsuji et al., 2016 | Scrophulariaceae *Mimulus aurantiacus* |  |  | florivory |
| Valdivia & Niemeyer, 2005 | Liliaceae *Alstroemeria umbellata* | *Aphis alstroemeriae* |  | florivory |
| Vallius & Salonen, 2006 | Orchidaceae *Platanthera bifolia* |  |  | folivory |
| Vazquez & Simberloff, 2004 | Liliaceae *Alstroemeria aurea,* Elaeocarpaceae *Aristotelia chilensis,* Asclepiadaceae *Cynanchum diemii,* Grossulariaceae *Ribes magellanicum* | *Bos taurus* |  | grazing |
| Vulliamy, Potts & Willmer, 2006 |  | *Bos taurus* |  | grazing |
| Zangerl & Berenbaum, 2009 | Apiaceae *Pastinaca sativa* | *Depressaria pastinacella* |  | florivory |
| Malo, Leirana-Alcocer & Parra-Tabla, 2001 | Orchidaceae *Myrmecophila tibicinis* |  |  | florivory |
| Strauss, Conner & Rush, 1996 | Brassicaceae *Raphanus raphanistrum* | *Pieris rapae* |  | folivory |

Adler LS. 2000. Alkaloid uptake increases fitness in a hemiparasitic plant via reduced herbivory and increased pollination. The American Naturalist 156:92–99. DOI: 10.1086/303374.

Adler LS, Karban R, Strauss SY. 2001. Direct and indirect effects of alkaloids on plant fitness Via herbivory and pollination. Ecology 82:2032–2044.

Agrawal AA, Rudgers JA, Botsford LW, Cutler D, Gorin JB, Lundquist CJ, Spitzer BW, Swann AL. 2000. Benefits and constraints on plant defense against herbivores: spines influence the legitimate and illegitimate flower visitors of yellow star thistle , Centaurea solstitialis L. (Asteraceae). The Southwestern Naturalist 45:1–5. DOI: 10.2307/3672545.

Åhman I, Lehrman A, Ekbom B. 2009. Impact of herbivory and pollination on performance and competitive ability of oilseed rape transformed for pollen beetle resistance. Arthropod-Plant Interactions 3:105–113. DOI: 10.1007/s11829-009-9061-z.

Arceo-Gómez G, Parra-Tabla V, Navarro J. 2009. Changes in sexual expression as result of defoliation and environment in a monoecious shrub in Mexico: implications for pollination. Biotropica 41:435–441. DOI: 10.1111/j.1744-7429.2009.00502.x.

Aschero V, Vázquez DP. 2009. Habitat protection, cattle grazing and density-dependent reproduction in a desert tree. Austral Ecology 34:901–907. DOI: 10.1111/j.1442-9993.2009.01997.x.

Barber NA, Milano NJ, Kiers ET, Theis N, Bartolo V, Hazzard R V., Adler LS. 2015. Root herbivory indirectly affects above- and below-ground community members and directly reduces plant performance. Journal of Ecology 103:1509–1518. DOI: 10.1111/1365-2745.12464.

Benning JW, Moeller DA. 2019. Maladaptation beyond a geographic range limit driven by antagonistic and mutualistic biotic interactions across an abiotic gradient. Evolution:1–16. DOI: 10.1111/evo.13836.

Botto-Mahan C, Ramírez PA, Ossa CG, Medel R, Ojeda-Camacho M, González A V. 2011. Floral herbivory affects female reproductive success and pollinator visitation in the perennial herb Alstroemeria ligtu (Alstroemeriaceae). International Journal of Plant Sciences 172:1130–1136. DOI: 10.1086/662029.

Brody AK, Irwin RE. 2012. When resources don’t rescue: flowering phenology and species interactions affect compensation to herbivory in Ipomopsis aggregata. Oikos 121:1424–1434. DOI: 10.1111/j.1600-0706.2012.20458.x.

Buchanan AL. 2014. Effects of damage and pollination on sexual and asexual reproduction in a flowering clonal plant. Plant Ecology 216:273–282. DOI: 10.1007/s11258-014-0434-8.

Caballero P, Ossa CG, Gonzáles WL, González-Browne C, Astorga G, Murúa MM, Medel R. 2013. Testing non-additive effects of nectar-robbing ants and hummingbird pollination on the reproductive success of a parasitic plant. Plant Ecology 214:633–640. DOI: 10.1007/s11258-013-0195-9.

Canela MBF, Sazima M. 2003. Florivory by the crab Armases angustipes (Grapsidae) influences hummingbird visits to Aechmea pectinata (Bromeliaceae). Biotropica 35:289–294. DOI: 10.1111/j.1744-7429.2003.tb00287.x.

Cardel YJ, Koptur S. 2010. Effects of florivory on the pollination of flowers: an experimental field study with a perennial plant. International Journal of Plant Sciences 171:283–292. DOI: 10.1086/650154.

Cares-Suárez R, Poch T, Acevedo RF, Acosta-Bravo I, Pimentel C, Espinoza C, Cares RA, Muñoz P, González A V, Botto-Mahan C. 2011. Do pollinators respond in a dose-dependent manner to flower herbivory?: an experimental assessment in Loasa tricolor (Loasaceae). Gayana. Botánica 68:176–181. DOI: 10.4067/S0717-66432011000200007.

Cariveau DP, Norton AP. 2014. Direct effects of a biocontrol agent are greater than indirect effects through flower visitors for the alien plant Dalmatian toadflax (Linaria dalmatica: Scrophulariaceae). Biological Invasions 16:1951–1960. DOI: 10.1007/s10530-013-0638-1.

Chalcoff VR, Lescano MN, Devegili AM. 2019. Do novel interactions with local fauna have reproductive consequences for exotic plants? A case study with thistles, ants, aphids, and pollinators. Plant Ecology 220:125–134. DOI: 10.1007/s11258-019-00907-2.

Cunningham SA. 1995. Ecological constraints on fruit initiation by Calyptrogyne ghiesberghtiana (Arecaceae): floral herbivory, pollen availability, and visitation by pollinating bats. American Journal of Botany 82:1527–1536.

Danderson CA, Molano-Flores B. 2010. Effects of herbivory and inflorescence size on insect visitation to Eryngium yuccifolium (Apiaceae) a prairie plant. The American Midland Naturalist 163:234–246. DOI: 10.1674/0003-0031-163.1.234.

Enri SR, Probo M, Farruggia A, Lanore L, Blanchetete A, Dumont B. 2017. A biodiversity-friendly rotational grazing system enhancing flower-visiting insect assemblages while maintaining animal and grassland productivity. Agriculture, Ecosystems and Environment 241:1–10. DOI: 10.1016/j.agee.2017.02.030.

Garrido P, Mårell A, Öckinger E, Skarin A, Jansson A, Thulin CG. 2019. Experimental rewilding enhances grassland functional composition and pollinator habitat use. Journal of Applied Ecology 56:946–955. DOI: 10.1111/1365-2664.13338.

Hambäck PA. 2001. Direct and indirect effects of herbivory: Feeding by spittlebugs affects pollinator visitation rates and seedset of rudbeckia hirta. Ecoscience 8:45–50. DOI: 10.1080/11956860.2001.11682629.

Hladun KR, Adler LS. 2009. Influence of leaf herbivory, root herbivory, and pollination on plant performance in Cucurbita moschata. Ecological Entomology 34:144–152. DOI: 10.1111/j.1365-2311.2008.01060.x.

Juenger T, Bergelson J. 1997. Pollen and Resource Limitation of Compensation To Herbivory in Scarlet Gilia, Ipomopsis Aggregata. Ecology 78:1684–1695. DOI: 10.1890/0012-9658(1997)078[1684:PARLOC]2.0.CO;2.

Kelly D, Ladley JJ, Robertson AW, Crowfoot L. 2008. Flower predation by Zelleria maculata (Lepidoptera) on Peraxilla mistletoes: effects of latitude and fragmentation, and impact on fruit set. New Zealand Journal of Ecology 32:186–196.

Knight TM. 2004. The effects of herbivory and pollen limitation on a declining population of Trillium grandiflorum. Ecological Applications 14:915–928. DOI: 10.1890/03-5048.

Krupnick GA, Weis AE. 1999. The effect of floral herbivory on male and female reproductive success in Isomeris arborea. Ecology 80:135–149. DOI: 10.1890/0012-9658(1999)080[0135:TEOFHO]2.0.CO;2.

Krupnick GA, Weis AE, Campbell DR. 1999. The consequence of floral herbivory for pollinator service. Ecology 80:125–134.

Leavitt H, Robertson IC. 2006. Petal herbivory by chrysomelid beetles (Phyllotreta sp.) is detrimental to pollination and seed production in Lepidium papilliferum (Brassicaceae). Ecological Entomology 31:657–660. DOI: 10.1111/j.1365-2311.2006.00820.x.

Liao K, Gituru RW, Guo YH, Wang QF. 2013. Effects of floral herbivory on foraging behaviour of bumblebees and female reproductive success in Pedicularis gruina (Orobanchaceae). Flora: Morphology, Distribution, Functional Ecology of Plants 208:562–569. DOI: 10.1016/j.flora.2013.08.007.

Lohman DJ, Zangerl AR, Berenbaum MR. 1996. Impact of floral herbivory by parsnip webworm (Oecophoridae: Depressaria pastinacella Duponchel ) on pollination and fitness of wild parsnip (Apiaceae: Pastinaca sativa L.). The American Midland Naturalist 136:407–412.

Louthan A, Valencia E, Martins DJ, Guy T, Goheen J, Palmer T, Doak D. 2019. Large mammals generate both top-down effects and extended trophic cascades on floral-visitor assemblages. Journal of Tropical Ecology 35:185–198. DOI: 10.1017/S0266467419000142.

Lucas-Barbosa D, van Loon JJA, Gols R, van Beek TA, Dicke M. 2013. Reproductive escape: annual plant responds to butterfly eggs by accelerating seed production. Functional Ecology 27:245–254. DOI: 10.1111/1365-2435.12004.

Lucas-Barbosa D, Sun P, Hakman A, van Beek TA, van Loon JJA, Dicke M. 2016. Visual and odour cues: plant responses to pollination and herbivory affect the behaviour of flower visitors. Functional Ecology 30:431–441. DOI: 10.1111/1365-2435.12509.

Malo JE, Leirana-Alcocer J, Parra-Tabla V. 2001. Population fragmentation, florivory, and the effects of flower morphology alterations on the pollination success of Myrmecophila tibicinis (Orchidaceae). Biotropica 33:529–534. DOI: 10.1111/j.1744-7429.2001.tb00207.x.

McCall AC. 2010. Does dose-dependent petal damage affect pollen limitation in an annual plant? Botany 88:601–606. DOI: 10.1139/B10-032.

Missagia CCC, Alves MAS. 2017. Florivory and floral larceny by fly larvae decrease nectar availability and hummingbird foraging visits at Heliconia (Heliconiaceae) flowers. Biotropica 49:13–17. DOI: 10.1111/btp.12368.

Moranz RA, Fuhlendorf SD, Engle DM. 2014. Making sense of a prairie butterfly paradox: the effects of grazing, time since fire, and sampling period on regal fritillary abundance. Biological Conservation 173:32–41. DOI: 10.1016/j.biocon.2014.03.003.

Mothershead K, Marquis RJ. 2000. Fitness impact of herbivore through indirect effects on plant-pollinator interactions in Oenothera macrocarpa. Ecology 81:30–40. DOI: 10.1890/0012-9658(2000)081[0030:FIOHTI]2.0.CO;2.

Motzke I, Tscharntke T, Wanger TC, Klein AM. 2015. Pollination mitigates cucumber yield gaps more than pesticide and fertilizer use in tropical smallholder gardens. Journal of Applied Ecology 52:261–269. DOI: 10.1111/1365-2664.12357.

Munguía-Rosas MA, Arias LM, Jurado-Dzib SG, Mezeta-Cob CR, Parra-Tabla V. 2015. Effects of herbivores and pollinators on fruit yield and survival in a cleistogamous herb. Plant Ecology 216:517–525. DOI: 10.1007/s11258-015-0455-y.

Rusman Q, Poelman EH, Nowrin F, Polder G, Lucas-Barbosa D. 2019. Floral plasticity: Herbivore-species-specific-induced changes in flower traits with contrasting effects on pollinator visitation. Plant Cell and Environment 42:1882–1896. DOI: 10.1111/pce.13520.

Sasal Y, Farji-Brener A, Raffaele E. 2017. Fire modulates the effects of introduced ungulates on plant–insect interactions in a Patagonian temperate forest. Biological Invasions 19:2459–2475. DOI: 10.1007/s10530-017-1455-8.

Scopece G, Frachon L, Cozzolino S. 2019. Do native and invasive herbivores have an effect on Brassica rapa pollination? Plant Biology 21:927–934. DOI: 10.1111/plb.12985.

Sõber V, Moora M, Teder T. 2010. Florivores decrease pollinator visitation in a self-incompatible plant. Basic and Applied Ecology 11:669–675. DOI: 10.1016/j.baae.2010.09.006.

Sõber V, Teder T, Moora M. 2009. Contrasting effects of plant population size on florivory and pollination. Basic and Applied Ecology 10:737–744. DOI: 10.1016/j.baae.2009.06.003.

Soper Gorden NL, Adler LS. 2016. Florivory shapes both leaf and floral interactions. Ecosphere 7:1–15. DOI: 10.1002/ecs2.1326.

Strauss SY, Conner JK, Rush SL. 1996. Foliar Herbivory Affects Floral Characters and Plant Attractiveness to Pollinators: Implications for Male and Female Plant Fitness. The American Naturalist 147:1098–1107.

Strauss SY, Murch P. 2004. Towards an understanding of the mechanisms of tolerance: Compensating for herbivore damage by enhancing a mutualism. Ecological Entomology 29:234–239. DOI: 10.1111/j.0307-6946.2004.00587.x.

Suárez-Esteban A, Delibes M, Fedriani JM. 2014. Unpaved roads disrupt the effect of herbivores and pollinators on the reproduction of a dominant shrub. Basic and Applied Ecology 15:524–533. DOI: 10.1016/j.baae.2014.08.001.

Suárez LH, Gonzáles WL, Gianoli E. 2009. Foliar damage modifies floral attractiveness to pollinators in Alstroemeria exerens. Evolutionary Ecology 23:545–555. DOI: 10.1007/s10682-008-9254-4.

Sutter L, Albrecht M. 2016. Synergistic interactions of ecosystem services: pest control boosts crop yield increase through insect pollination. Proceedings of the Royal Society B. DOI: 10.1098/rspb.2015.2529.

Torres I, Salinas L, Lara C, Castillo-Guevara C. 2008. Antagonists and their effects in a hummingbird–plant interaction: Field experiments. Ecoscience 15:65–72. DOI: 10.2980/1195-6860(2008)15[65:AATEIA]2.0.CO;2.

Tsuji K, Dhami MK, Cross DJR, Rice CP, Romano NH, Fukami T. 2016. Florivory and pollinator visitation: A cautionary tale. AoB PLANTS 8. DOI: 10.1093/aobpla/plw036.

Valdivia CE, Niemeyer HM. 2005. Reduced maternal fecundity of the high Andean perennial herb Alstroemeria umbellata (Alstroemeriaceae) by aphid herbivory. New Zealand Journal of Ecology 29:321–324.

Vallius E, Salonen V. 2006. Allocation to reproduction following experimental defoliation in Platanthera bifolia (Orchidaceae). Plant Ecology 183:291–304. DOI: 10.1007/s11258-005-9040-0.

Vazquez DP, Simberloff D. 2004. Indirect Effects of an Introduced Ungulate on Pollination and Plant Reproduction. Ecological Monographs 74:281–308.

Vulliamy B, Potts SG, Willmer PG. 2006. The effects of cattle grazing on plant-pollinator communities in a fragmented Mediterranean landscape. Oikos 114:529–543. DOI: 10.1111/j.2006.0030-1299.14004.x.

Zangerl AR, Berenbaum MR. 2009. Effects of florivory on floral volatile emissions and pollination success in the wild parsnip. Arthropod-Plant Interactions 3:181–191. DOI: 10.1007/s11829-009-9071-x.
