# Supplementary material for: A systematic review of the direct and indirect effects of herbivory on plant reproduction mediated by pollination
Source: PeerJ. 2020 Jun 8;8:e9049. doi: 10.7717/peerj.9049 (PMC7289145; doi:10.7717/peerj.9049)
Supplement: Supplemental Information 7 — When no herbivores were examined, herbivory was mimicked through either artificial herbivory or application of chemicals. When no pollinators were examined, pollination was studied through supplemental hand pollination. Community refers to herbivory or pollination that was not restricted, that is the entire community or order (or family) of herbivores and pollinators had access to the plant. [file peerj-08-9049-s007.docx]

**Supplemental Table S3.** Counts of the number of studies that examined a given diversity of species for each of plants, herbivores, and pollinators. When no herbivores were examined, herbivory was mimicked through either artificial herbivory or application of chemicals. When no pollinators were examined, pollination was studied through supplemental hand pollination. Community refers to herbivory or pollination that was not restricted, that is the entire community or order (or family) of herbivores and pollinators had access to the plant.

|  | **Plants** | **Herbivores** | **Pollinators** |
| --- | --- | --- | --- |
| **One Species** | 52 | 26 | 4 |
| **Two Species** | 1 | 2 | 3 |
| **Three Species** | 0 | 1 | 1 |
| **Four Species** | 1 | 1 | 0 |
| **Six Species** | 0 | 1 | 0 |
| **Community** | 5 | 11 | 44 |
| **None** | 0 | 17 | 7 |
