# Supplementary material for: A systematic review of the direct and indirect effects of herbivory on plant reproduction mediated by pollination
Source: PeerJ. 2020 Jun 8;8:e9049. doi: 10.7717/peerj.9049 (PMC7289145; doi:10.7717/peerj.9049)
Supplement: Supplemental Information 8 — Totals and subtotals of the number of studies that examined each type of herbivory, response, and effect direction are also presented. [file peerj-08-9049-s008.docx]

**Supplemental Table S4.** The number of studies that found some negative, neutral, or positive effect of herbivory (florivory, folivory, grazing, open, root herbivory, or stem damage) on some aspect of floral expression, pollination, or seed production. Totals and subtotals of the number of studies that examined each type of herbivory, response, and effect direction are also presented.

| **Effect Direction** | **Floral Expression** | **Pollination** | **Seed Production** | **Total** |
| --- | --- | --- | --- | --- |
| **Florivory** |  |  |  |  |
| negative | 6 | 18 | 15 | 25 |
| neutral | 4 | 6 | 9 | 14 |
| positive | 0 | 1 | 6 | 7 |
| Subtotal | **9** | **22** | **19** | **29** |
| **Folivory** |  |  |  |  |
| negative | 7 | 5 | 6 | 13 |
| neutral | 8 | 6 | 6 | 13 |
| positive | 1 | 0 | 4 | 5 |
| Subtotal | **9** | **10** | **7** | **16** |
| **Grazing** |  |  |  |  |
| negative | 3 | 5 | 4 | 8 |
| neutral | 5 | 5 | 5 | 8 |
| positive | 3 | 3 | 1 | 4 |
| Subtotal | **8** | **9** | **7** | **12** |
| **Open** |  |  |  |  |
| negative | 0 | 0 | 2 | 2 |
| neutral | 0 | 1 | 1 | 2 |
| positive | 0 | 0 | 1 | 1 |
| Subtotal |  | **1** | **2** | **2** |
| **Root Herbivory** |  |  |  |  |
| negative | 2 | 1 | 1 | 2 |
| neutral | 3 | 2 | 2 | 3 |
| positive | 1 | 1 | 0 | 1 |
| Subtotal | **3** | **2** | **2** | **3** |
| **Stem Damage** |  |  |  |  |
| negative | 4 | 3 | 2 | 5 |
| neutral | 2 | 0 | 2 | 3 |
| positive | 1 | 1 | 0 | 1 |
| Subtotal | **4** | **3** | **3** | **5** |
|  |  |  |  |  |
| **Total** | **29** | **42** | **40** | **59** |
