# Supplementary material for: A systematic review of the direct and indirect effects of herbivory on plant reproduction mediated by pollination
Source: PeerJ. 2020 Jun 8;8:e9049. doi: 10.7717/peerj.9049 (PMC7289145; doi:10.7717/peerj.9049)
Supplement: Supplemental Information 9 [file peerj-08-9049-s009.docx]

| **Section/topic** | **#** | **Checklist item** | **Reported on page #** |
| --- | --- | --- | --- |
| **TITLE** | | |  |
| Title | 1 | Identify the report as a systematic review, meta-analysis, or both.  Text: A systematic review of the direct and indirect effects of herbivory on plant reproduction mediated by pollination: theories, methods, and challenges | 1 |
| **ABSTRACT** | | |  |
| Structured summary | 2 | Provide a structured summary including, as applicable: background; objectives; data sources; study eligibility criteria, participants, and interventions; study appraisal and synthesis methods; results; limitations; conclusions and implications of key findings; systematic review registration number.  Text: **Background.** Plant reproduction is influenced by the net outcome of plant-herbivore and plant-pollinator interactions. While both herbivore impacts and pollinator impacts on plant reproduction have been widely studied, few studies examine them in concert.  **Methodology.** Here, we review the contemporary literature that examines the net outcomes of herbivory and pollination on plant reproduction and the impacts of herbivores on pollination through damage to shared host plants using systematic review tools. The direct or indirect effects of herbivores on floral tissue and reported mechanisms were compiled including the taxonomic breadth of herbivores, plants, and pollinators.  **Results.** A total of 4304 studies were examined producing 61 relevant studies for synthesis that reported both pollinator and herbivore measures. A total of 53% of studies examined the impact of direct damage to floral tissue through partial florivory while 35% of studies also examined the impact of vegetative damage on pollination through folivory, root herbivory, and stem damage. Only 4 studies examined the effects of both direct and indirect damage to pollination outcomes within the same study.  **Conclusions.** It is not unreasonable to assume that plants often sustain simultaneous forms of damage to different tissues and that the net effects can be assessed through differences in reproductive output. Further research that controls for other relative drivers of reproductive output but examines more than one pathway of damage simultaneously will inform our understanding of the mechanistic relevance of herbivore impacts on pollination and also highlight interactions between herbivores and pollinators through plants. It is clear that herbivory can impact plant fitness through pollination; however, the relative importance of direct and indirect damage to floral tissue on plant reproduction is still largely unknown. | 1 |
| **INTRODUCTION** | | |  |
| Rationale | 3 | Describe the rationale for the review in the context of what is already known.  Text: …herbivory and pollination also interact in more subtle and indirect ways. Negative impacts to pollinators can amplify the negative effects of herbivores on plant fitness by reducing both potential seed set (e.g. number of flowers available to set seed) (Strauss, Conner & Rush, 1996; Hambäck, 2001; Rusman et al., 2019) and actual seed set (i.e. flowers are not all pollinated due to decreased pollinator visitation) (Adler, Karban & Strauss, 2001; Benning & Moeller, 2019). Therefore, when the net outcome of herbivory on plants is examined, pollination must also be considered. In this systematic review, we have outlined a conceptual framework (Fig. 1) to illustrate the direct and indirect ways in which the negative effects of herbivory can both directly and indirectly effect plant fitness via animal pollinators and pollination. | 2 |
| Objectives | 4 | Provide an explicit statement of questions being addressed with reference to participants, interventions, comparisons, outcomes, and study design (PICOS).  Text: In this systematic review, we synthesize the contemporary literature on herbivore-plant-pollinator interactions with a specific focus on studies that examined the joint impact of herbivores and pollinators on plant reproduction or the impact of herbivores on pollination using the mechanistic pathways proposed in our conceptual framework (Fig. 1). The frequency of mechanisms tested and the frequency that direct vs indirect floral damage pathways are contrasted is important to both ecology and evolution. This includes examining the diversity of types of damage—both the tissue targeted and the taxa causing the damage. Finally, we examine how each mechanism is tested. | 5 |
| **METHODS** | | |  |
| Protocol and registration | 5 | Indicate if a review protocol exists, if and where it can be accessed (e.g., Web address), and, if available, provide registration information including registration number.  Text: no review protocol was registered | NA |
| Eligibility criteria | 6 | Specify study characteristics (e.g., PICOS, length of follow-up) and report characteristics (e.g., years considered, language, publication status) used as criteria for eligibility, giving rationale.  Text: Papers had to meet the criteria that they directly tested the impact of herbivory on animal-mediated pollination. The indirect effect of herbivores on pollinators or the indirect effect of herbivores on plants via pollinators must have been reported to be included in this synthesis (e.g. through measuring pollen deposition or comparing open pollination to supplementary hand pollination) | 5-6 |
| Information sources | 7 | Describe all information sources (e.g., databases with dates of coverage, contact with study authors to identify additional studies) in the search and date last searched.  Text: A search for papers that examine the impact of herbivores on pollinators or the pollination of plants in October 2019 using Web of Science… These papers ranged from 1995 to 2019 spanning 18 different countries | 5-6 |
| Search | 8 | Present full electronic search strategy for at least one database, including any limits used, such that it could be repeated.  Text: A search for papers that examine the impact of herbivores on pollinators or the pollination of plants in October 2019 using Web of Science and the search terms “herbivor* AND pollinat*”, “floriv*”, “foliv* AND pollinat*”, “herbivor* AND flower*”, and “foliv* AND flower” was conducted… Papers had to meet the criteria that they directly tested the impact of herbivory on animal-mediated pollination. The indirect effect of herbivores on pollinators or the indirect effect of herbivores on plants via pollinators must have been reported to be included in this synthesis (e.g. through measuring pollen deposition or comparing open pollination to supplementary hand pollination) ... Papers that were excluded were reviews and descriptions of the natural history of plants or animals (including diet). Studies were also excluded if they examined the impact of herbivores on plants but not pollination. | 5-6 |
| Study selection | 9 | State the process for selecting studies (i.e., screening, eligibility, included in systematic review, and, if applicable, included in the meta-analysis).  Text: Papers had to meet the criteria that they directly tested the impact of herbivory on animal-mediated pollination. The indirect effect of herbivores on pollinators or the indirect effect of herbivores on plants via pollinators must have been reported to be included in this synthesis (e.g. through measuring pollen deposition or comparing open pollination to supplementary hand pollination) ... Papers that were excluded were reviews and descriptions of the natural history of plants or animals (including diet). Studies were also excluded if they examined the impact of herbivores on plants but not pollination. | 5-6 |
| Data collection process | 10 | Describe method of data extraction from reports (e.g., piloted forms, independently, in duplicate) and any processes for obtaining and confirming data from investigators.  Text: Data extracted included the physical location of all study sites and the taxa examined, as well as the analyses performed (including type of herbivory, response variable, and general direction of effect each variable had on each response) and the general structure of the experimental design. | 6 |
| Data items | 11 | List and define all variables for which data were sought (e.g., PICOS, funding sources) and any assumptions and simplifications made.  Text: Data extracted included the physical location of all study sites and the taxa examined, as well as the analyses performed (including type of herbivory, response variable, and general direction of effect each variable had on each response) and the general structure of the experimental design. | 6, Supplemental File Metadata |
| Risk of bias in individual studies | 12 | Describe methods used for assessing risk of bias of individual studies (including specification of whether this was done at the study or outcome level), and how this information is to be used in any data synthesis. | NA |
| Summary measures | 13 | State the principal summary measures (e.g., risk ratio, difference in means). | NA |
| Synthesis of results | 14 | Describe the methods of handling data and combining results of studies, if done, including measures of consistency (e.g., I^2^) for each meta-analysis. | NA |

Page 1 of 2

| **Section/topic** | **#** | **Checklist item** | **Reported on page #** |
| --- | --- | --- | --- |
| Risk of bias across studies | 15 | Specify any assessment of risk of bias that may affect the cumulative evidence (e.g., publication bias, selective reporting within studies). | NA |
| Additional analyses | 16 | Describe methods of additional analyses (e.g., sensitivity or subgroup analyses, meta-regression), if done, indicating which were pre-specified. | NA |
| **RESULTS** | | |  |
| Study selection | 17 | Give numbers of studies screened, assessed for eligibility, and included in the review, with reasons for exclusions at each stage, ideally with a flow diagram.  Text: This resulted in 3681 papers… In total, 59 papers met all criteria to be included in the final analysis. Fig. 1 | 3-4, Fig. 1 |
| Study characteristics | 18 | For each study, present characteristics for which data were extracted (e.g., study size, PICOS, follow-up period) and provide the citations.  Text: See Supplemental Table S1 & S2 | Supplemental Tables S1 & S2 |
| Risk of bias within studies | 19 | Present data on risk of bias of each study and, if available, any outcome level assessment (see item 12). | NA |
| Results of individual studies | 20 | For all outcomes considered (benefits or harms), present, for each study: (a) simple summary data for each intervention group (b) effect estimates and confidence intervals, ideally with a forest plot. | NA |
| Synthesis of results | 21 | Present results of each meta-analysis done, including confidence intervals and measures of consistency. | NA |
| Risk of bias across studies | 22 | Present results of any assessment of risk of bias across studies (see Item 15). | NA |
| Additional analysis | 23 | Give results of additional analyses, if done (e.g., sensitivity or subgroup analyses, meta-regression [see Item 16]). | NA |
| **DISCUSSION** | | |  |
| Summary of evidence | 24 | Summarize the main findings including the strength of evidence for each main outcome; consider their relevance to key groups (e.g., healthcare providers, users, and policy makers).  Sample Text: The overall impact of the community of herbivores can impact plant resource allocation, compensation and therefore pollination. However, the effects of herbivores on pollination are frequently examined through a single herbivore and feeding strategy. Interactions do not exist in isolation: plants generally experience damage from multiple herbivorous taxa (often including both insects and mammals) and damage to multiple vegetative and floral tissues (Maddox & Root, 1987; Farré-Armengol et al., 2015; Wise & Rausher, 2016). While examining the impact of individual herbivores is important, in order to study the resilience and community dynamics of plants and pollinators in relation to herbivores, it is important to consider multiple herbivores as well as both direct and indirect impacts on floral tissue, following the conceptual framework outlined in Fig. 1. The indirect effect of herbivores on pollinators can mediate co-evolutionary processes between plants and herbivores and plants and pollinators. Therefore, understanding how different types and forms of herbivory impact pollinators is important for understanding both ecological and evolutionary stability. | 6-9 |
| Limitations | 25 | Discuss limitations at study and outcome level (e.g., risk of bias), and at review-level (e.g., incomplete retrieval of identified research, reporting bias).  Sample Text: the sample size of studies that examine any other form of herbivory is particularly low | 6-9 |
| Conclusions | 26 | Provide a general interpretation of the results in the context of other evidence, and implications for future research.  Text: Both direct and indirect damage to floral tissue can impact pollination and plant reproduction. However, direct and indirect damage to floral tissue is rarely examined in concert. The relative importance of the direct and indirect mechanisms and synergistic effects have important implications for ecological resilience and stability in evolutionary processes. However, this relative importance is almost never examined with the focus lying on each type individually. The collection of herbivores that interact with plants can include species that feed on all types of tissue either simultaneously or temporally separated that the plant then integrates into growth, allocation, defense, or phenology. This in turn can impact pollinators and pollination, making these two plant-animal interactions intimately linked. | 9 |
| **FUNDING** | | |  |
| Funding | 27 | Describe sources of funding for the systematic review and other support (e.g., supply of data); role of funders for the systematic review.  Text: CJL was funded by an NSERC DG. SH was funded by York University. | 9 |

*From:*  Moher D, Liberati A, Tetzlaff J, Altman DG, The PRISMA Group (2009). Preferred Reporting Items for Systematic Reviews and Meta-Analyses: The PRISMA Statement. PLoS Med 6(7): e1000097. doi:10.1371/journal.pmed1000097

For more information, visit: **www.prisma-statement.org**.

Page 2 of 2
